# A high-resolution 4D terrestrial laser scan dataset of the Kijkduin beach-dune system, The Netherlands

**DOI:** 10.1038/s41597-022-01291-9

**Published:** 2022-04-28

**Authors:** Sander Vos, Katharina Anders, Mieke Kuschnerus, Roderik Lindenbergh, Bernhard Höfle, Stefan Aarninkhof, Sierd de Vries

**Affiliations:** 1 Department of Hydraulic Engineering, Delft University of Technology, Stevinweg 1, 2628 CN Delft, The Netherlands; 2 Baars-CIPRO, Hoofdweg 16a, 1175 LA Lijnden, The Netherlands; 3 3D Geo Research Group, Institute of Geography, Heidelberg University, Im Neuenheimer Feld 368, 69120 Heidelberg, Germany; 4 Department of Geoscience and Remote Sensing, Delft University of Technology, Stevinweg 1, 2628 CN Delft, The Netherlands

**Keywords:** Environmental impact, Geomorphology, Civil engineering

## Abstract

Sandy coasts form the interface between land and sea and their morphologies are highly dynamic. A combination of human and natural forcing results in morphologic changes affecting both nature values and coastal safety. Terrestrial laser scanning (TLS) is a technique enabling near-continuous monitoring of the changing morphology of a sandy beach-dune system with centimetre-order accuracy. In Kijkduin, The Netherlands, a laser scanner sampled one kilometre of coast at hourly intervals for about six months. This resulted in over 4,000 consecutive topographic scans of around one million points each, at decimetre-order point spacing. Analysis of the resulting dataset will offer new insights into the morphological behaviour of the beach-dune system at hourly to monthly time scales, ultimately increasing our fundamental scientific understanding of these complex geographic systems. It further provides the basis for developing novel algorithms to extract morphodynamic and geodetic information from this unique 4D spatiotemporal dataset. Finally, experiences from this TLS setup support the development of improved near-continuous 3D observation of both natural and anthropogenic scenes in general.

## Abstract (summary)

**Table:** 

Measurement(s)	Beach/dune height and surface properties
Technology Type(s)	Terrestial laser scanner
Sample Characteristic - Organism	Sandy beach-dune area
Sample Characteristic - Environment	Beach/dune area
Sample Characteristic - Location	Kijkduin, The Netherlands

## Background & Summary

Sandy coasts constitute about one third of all coasts in the world and their morphologies are highly dynamic in nature^1^. Humans have populated these areas for millennia^2^ and it is estimated that nowadays more than 40% of the world population lives within 100 kilometres from the shore^3,4^, a percentage which is expected to increase further^5^. This increasing coastal population has a large influence on coastal systems^6,7^ due to loss and disturbance of fragile ecosystems^8,9^, while sea level rise and changing weather patterns, due to climate change, add additional pressure to sandy coasts^10–13^. Presently, around 82,500 kilometres of sandy coasts worldwide show retreating coastlines^1^ amounting to an area^14^ of about 28,000 km2 which can be linked to human activities^15–17^.

In defence of the coast, hard structures like dikes, sea-walls and groins have been added at numerous places with varying effectiveness^18^. Sandy coastal adaptations with a larger degree of natural dynamics have been advocated as an alternative in recent years^19–21^. Such soft interventions enable flexible maintenance and easier coastal adaptation to predicted climate change. To fully utilize these adaptations, a deeper understanding of the complex geographic system of natural and artificial sandy coasts is needed. Monitoring of dynamic coastal processes is an essential part to achieve such understanding.

**Fig. 1:**
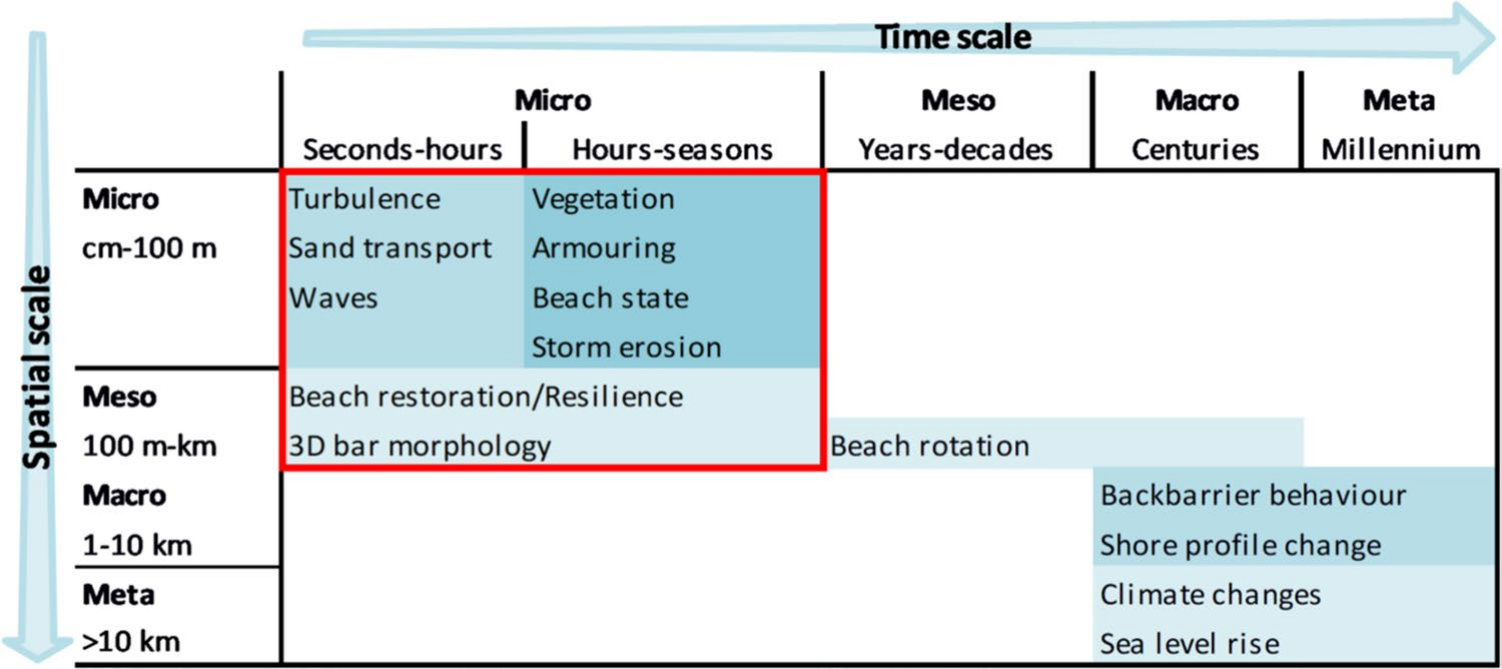
Overview of coastal processes at various spatiotemporal scales according to the Coastal Tract concept (modified from^68^). Processes within the red box can be monitored with near-continuous terrestrial laser scanning.

Coastal processes and associated morphological changes occur at multiple spatiotemporal scales ranging from centimetres to (many) kilometres and seconds to millennia (Fig. 1), which are interlinked and acting upon each other. The wide range of interacting spatial and temporal scales imposes a challenge to obtain suitable observations for monitoring. Numerous techniques are available to provide observations targeted to specific processes or morphodynamic properties (see literature overviews^22–25^), but few techniques allow measuring at various scales without extensive, time-consuming field campaigns.

Near-continuous terrestrial laser scanning (TLS) has recently become available to monitor spatiotemporal processes with the development of programmable accurate long-range terrestrial laser scanners^26^. Over the past years, permanent setups of these instruments have been utilised in so-called permanent laser scanning (PLS) around the world to acquire topographic representations of landslides^27^, vegetation^28^ and sandy/rocky coasts^22,29–31^, and to capture and investigate dynamics of these natural landscapes.

To improve our knowledge of the complex multi-process setting of sandy beaches and dunes, a PLS system has been set up at the beach-dune system of Kijkduin, The Netherlands^32^. The objective of this near-continuous 3D observation system and acquired data is to increase knowledge on the variability of coastal morphology and its resilience under the influence of anthropogenic modifications and natural dynamics, as well as to advance the methodology of permanent laser scanning in coastal monitoring towards operational applicability.

**Fig. 2:**
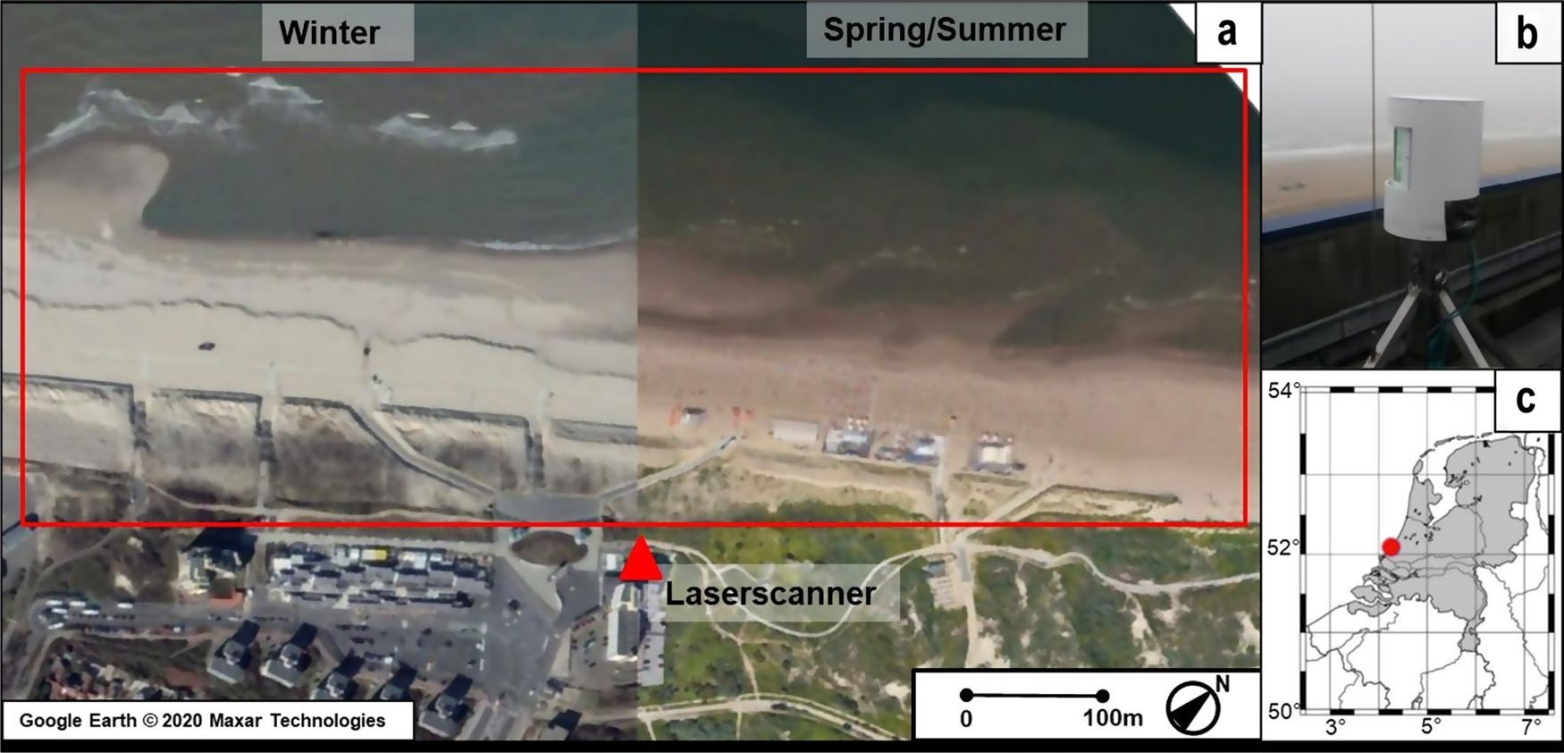
Overview of the study site. (**a**) Aerial photo of the winter and spring-summer beach-dune area (with beach pavilions in the spring-summer period) and the scan area within the red rectangle. (**b**) The laser scanner on a hotel next to the beach (indicated by a red triangle in **a**). (**c**) The location of Kijkduin (52.07°N, 4.22°E) in The Netherlands.

A terrestrial laser scanner was mounted in a permanent setup on top of a hotel at about 38 m height above mean sea level overlooking the beach and dunes at the coast of Kijkduin, The Netherlands (Fig. 2). The dynamic morphology of the coastal area at Kijkduin is influenced by both environmental and anthropogenic forces and is not reinforced with hard structures. The beach-dune area was scanned hourly during a six-months period in the winter-spring season of 2016/2017, generating a time series of 4,082 3D point clouds.

The laser scanning data in Kijkduin is georeferenced and checked against both RTK-GNSS (Real time kinematic Global Navigation Satellite System) measurements and airborne laser scanning (ALS) data of fixed objects and the beach-dune area. A data consistency check has been performed by assessing positions and orientations of fixed objects and surfaces in the point cloud scene over time^33,34^ (see also Section on technical validation).

Each of the 4,082 hourly point clouds of the beach-dune system at Kijkduin contains between one and ten million 3D points depending on the weather conditions and scan resolution. Additional attributes, such as the laser return intensity, are available for each individual 3D point record (see section Data Records).

The PLS dataset is valuable for studying hydrodynamics, morphology, aeolian transport, vegetation and the impact of anthropogenic behaviour. High-frequency measurements of the beach and dune surface are expected to provide insight on aeolian sand transport towards the coast during low tides. Over the full observational period of six months, processes can be characterized in terms of their variability, return frequency, and overall contribution to topography. Similarly, anthropogenic modifications can be timed and assessed for their effect on the long-term status of the beach-dune environment. In addition to coastal monitoring, the 4D point cloud dataset is valuable for investigating characteristics of near-continuous laser scanning and for algorithm development for improved change detection and characterization in time series of 3D point clouds. Methodology of 3D change analysis has only been recently starting to incorporate the full temporal information of such 4D point cloud data. The availability of a large PLS dataset may foster ongoing research for improved 4D change extraction. We provide some insights on analyses making use of the full time series information in the section Usage Notes.

## Methods

The 4D point cloud dataset was obtained with an eye-safe (class 1^35^) Riegl VZ-2000 laser scanner^36^ operating at a wavelength of 1,550 nm. The instrument was positioned on top of NH Hotel Atlantic in Kijkduin overlooking the beach (see Fig. 2) on a four-legged iron pole which was cross-braced to minimize horizontal displacements. The laser scanner was protected by a self-designed double PVC (Polyvinyl Chloride) housing to avoid fouling and temperature extremes potentially affecting the laser scanner.

**Fig. 3:**
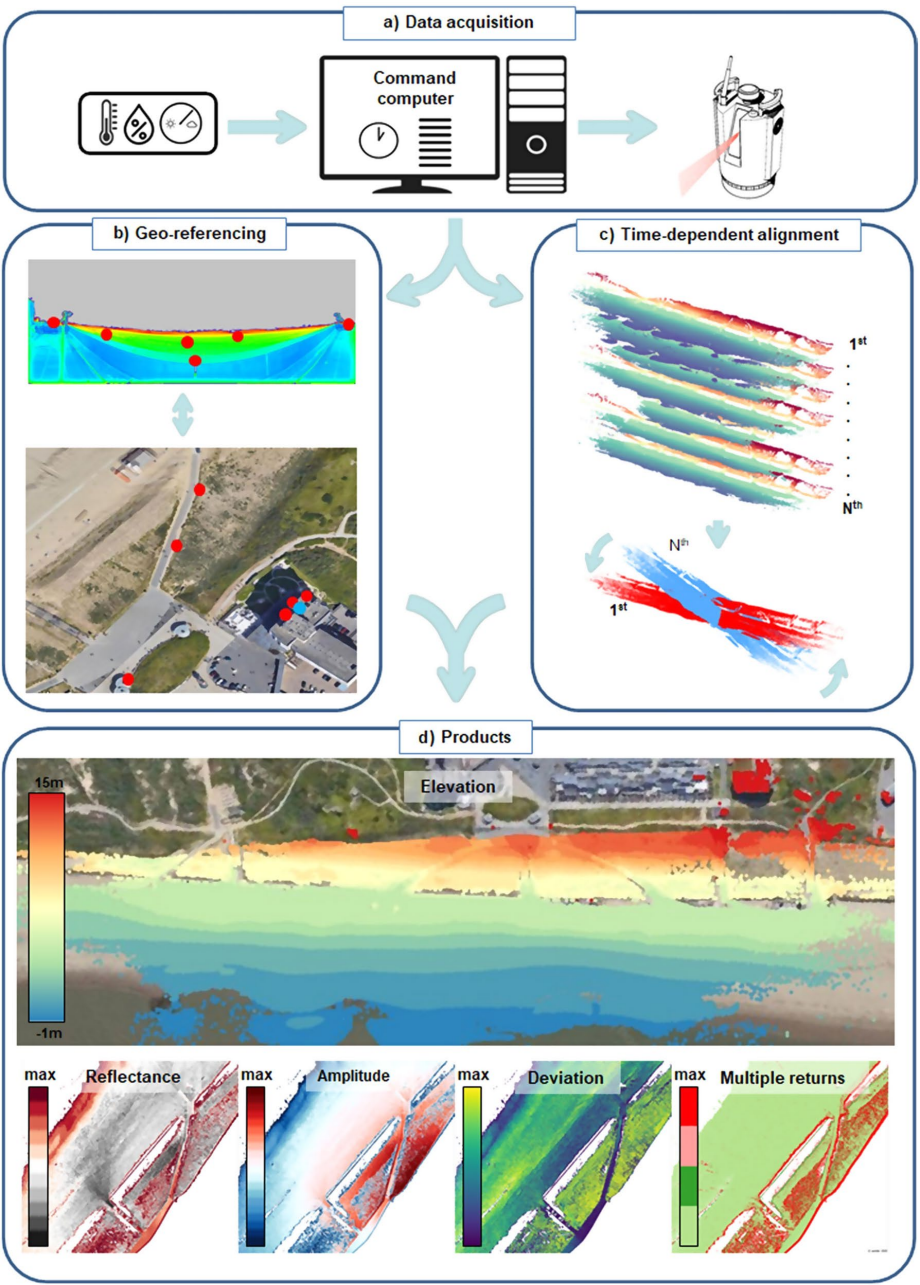
Schematic overview of the laser scan acquisition, processing and products. (**a**) Laser scans were initiated by a time-scheduled command computer with online weather information. (**b**) A global geo-reference transformation matrix was determined by matching reflectors (red dots, (**b**) upper image) in a scan of the laser scanner (blue dot) with GNSS measured reflectors in real space (**b**, lower image). (**c**) A time-dependent fine alignment transformation matrix for each epoch was obtained by comparing each N^th^ scan (upper figure **c**) with the first scan of the acquisition period (lower figure **c**). (**d**) Application of both transformations to a scan results in various georeferenced products.

The acquisition and processing scheme of the 4D point cloud is visualized in Fig. 3. Hourly scans were initiated with a command computer by providing the laser scanner with online weather information^37^. Atmospheric temperature, pressure and relative humidity were uploaded and used internally by the instrument to account for varying atmospheric influence on the time-of-flight range measurements.

The 3D point measurements were acquired in polar coordinates in the manufacturer’s proprietary format and converted to local Cartesian coordinates with the laser scanner sensor as origin (RivLib library^38^ [version 2.5.9]]). To reduce data volumes in the less relevant area near the laser scanner, each point cloud was subsampled to a minimum point spacing of 1 cm. Therein, all points with 1 cm distance around a randomly picked point are flagged until all points are either picked or flagged, and flagged points are deleted^39^. Point clouds are finally converted to losslessly compressed LAZ format^40^, in which coordinates are stored with millimetre precision. No other filtering of points or thresholding of any point attributes was applied in order to preserve all original measurements.

To improve the alignment accuracy between different point cloud epochs, a time-dependent alignment was derived for individual scans in the time series. For this, rigid (rotation/translation) transformation matrices were determined via the Iterative Closest Point (ICP) method^41^ using stable planar surfaces in the scene to align each epoch to the first in the series of scans (i.e., Nov 11^th^ 2017, 20:00).

Additionally, six circular reflector targets with five-centimetre diameter were spread out over a 200-degree horizontal field of view and were measured with a GNSS sensor at the beginning of the fieldwork. These measurements were used to obtain a rigid global transformation matrix^32^ to convert the local Cartesian coordinates into the Dutch National coordinate system (RD-NAP^42^). The matrix was calculated once at the beginning of the field campaign with the proprietary software Riscan Pro^43^ and is provided with the dataset.

Additional instrument data, consisting of 1 Hz inclination (pitch and roll) values during acquisition, is provided separately with each point cloud epoch. All data is available via the Pangaea data repository^44^.

## Data Records

**Fig. 4:**
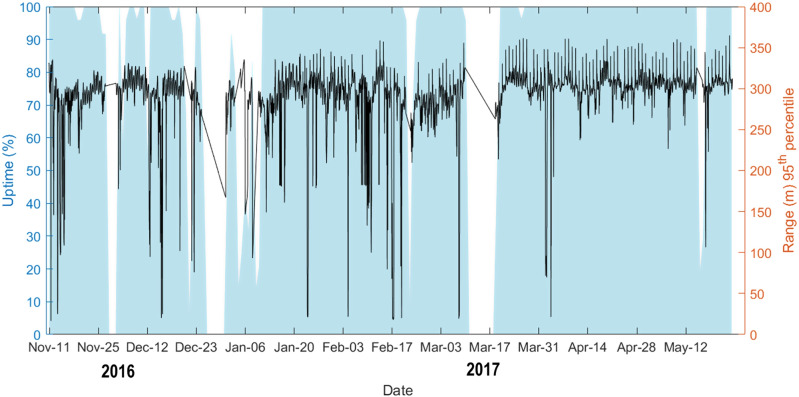
Daily uptime and scan range of the laser scanner. The uptime is indicated by the blue area and the range by the black line. The uptime (0–100%) is derived from the number of scans per day (reference is once per hour) while the scan range is expressed by the 95^th^ percentile of range values in the point cloud of each scan.

The 4D point cloud dataset^44^ contains 4,082 point clouds acquired hourly during 190 days between November 11^th^ 2016, 20:00 and May 26^th^ 2017, 08:00, covering most of the meteorological winter/spring season. Average daily uptime (i.e., time available for scanning per day) of the laser scanner in this period was about 85%. Figure 4 shows the daily uptime and scan range of the laser scanner (expressed as the 95^th^ percentile of measurement ranges [in metres]). High range values typically correspond to scan acquisition under clear weather conditions, whereas low values are associated with high humidity in the form of clouds, mist or rain that reduce the achievable scan range. Uptime at the end of 2016 and in March 2017 was low due to power outages, scheduler program bugs, and limited access to the site during the Christmas holiday season.

Scans were acquired with a pulse repetition of 100 kHz and a maximum range of 1,800 m to obtain good scan coverage in the outer ranges. The field of view was about 245° horizontally with a scan angle resolution of 0.05° horizontally and vertically.

Additional high-resolution scans (0.013° resolution) were acquired around noon on a daily basis starting from January 22^nd^ 2017. Typical scan duration was about 4 minutes for the normal resolution scan and 20 minutes for the high-resolution scan. Given these scan durations, it can, to some extent be assumed that weather is stable throughout the scans. Still, it may occasionally happen that heavy rain starts or stops during scanning. Such scans could be identified by an increase of noise, as some individual laser beams will be reflected by rain drops.

**Fig. 5:**
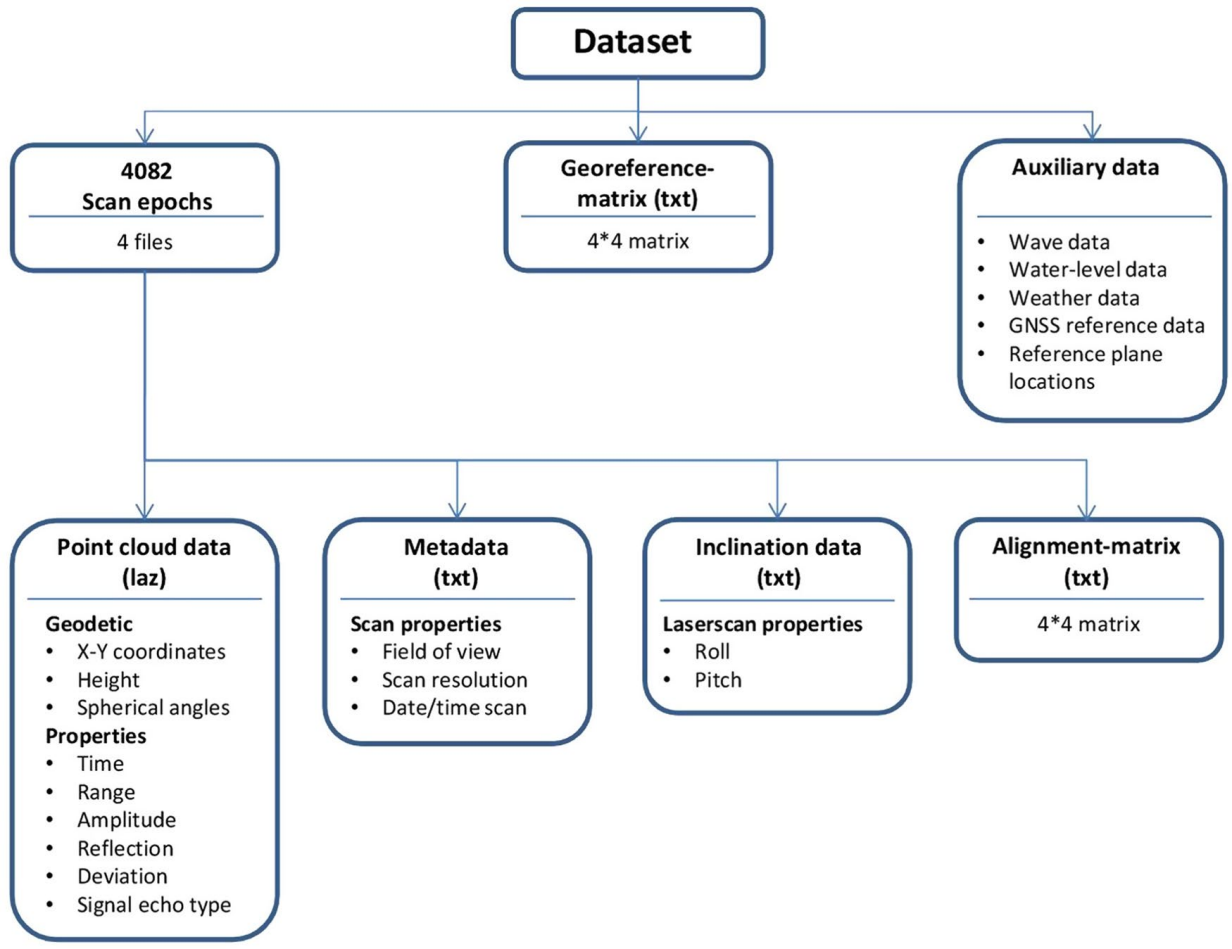
Visual representation of the laser scan dataset with supplementary data.

The structure of the scan dataset is visually represented in Fig. 5. One global transformation matrix is available for the georeferencing of all scans from local coordinates to Dutch national coordinates (RD-NAP). For each individual scan, four files are available containing point cloud data, metadata on acquisition settings, scanner inclination data (roll and pitch of the internal inclination sensor) and a rigid transformation matrix for time-dependent alignment. The time-dependent alignment matrix has to be applied before the global transformation matrix to obtain a correctly aligned and georeferenced point cloud.

Individual point clouds contain about one million points (median 1,063,034) for hourly scans and up to 11 million points (median 11,269,352) for daily high-resolution scans, each depending on weather and tidal conditions and scanner settings. Point information consists of the 3D coordinate and laser pulse information, which are amplitude, reflectance, shape deviation and echo types (see Fig. 5). The returned signal amplitude depends on the power of the emitted signal, on the range, and on the target reflectance. The reflectance attribute of each point is a range-corrected amplitude internally calculated by the scanner based on calibration of the manufacturer. The reflectance (here defined as by Riegl^45^ and not the target surface reflectance in the SI sense) is influenced by the target geometry, material, wetness, size in relation to the incoming laser beam footprint and incidence angle of the laser beam^46^. The deviation of the returned signal from the emitted pulse shape is dependent on the local surface structure, such as roughness and the incidence angle^47^. The signal echo type describes the recorded position of the returned signal from a single laser pulse. The return signal consists of a single or of multiple echo(es). Multiple echoes occur when the laser beam hits more than one target surface, for example where the laser beam penetrates vegetation on the dunes. The echo type returns for a laser pulse may be single, first, interior or last^48^.

The metadata for each epoch contains information about the scan pattern, i.e. field of view of the scan and vertical and horizontal angular resolution and the start date and time of the scan. A separate file is provided for each scan with inclination data recorded by the internal sensor, which contains the 1 Hz roll and pitch angles of the instrument during acquisition of the respective point cloud.

**Fig. 6:**
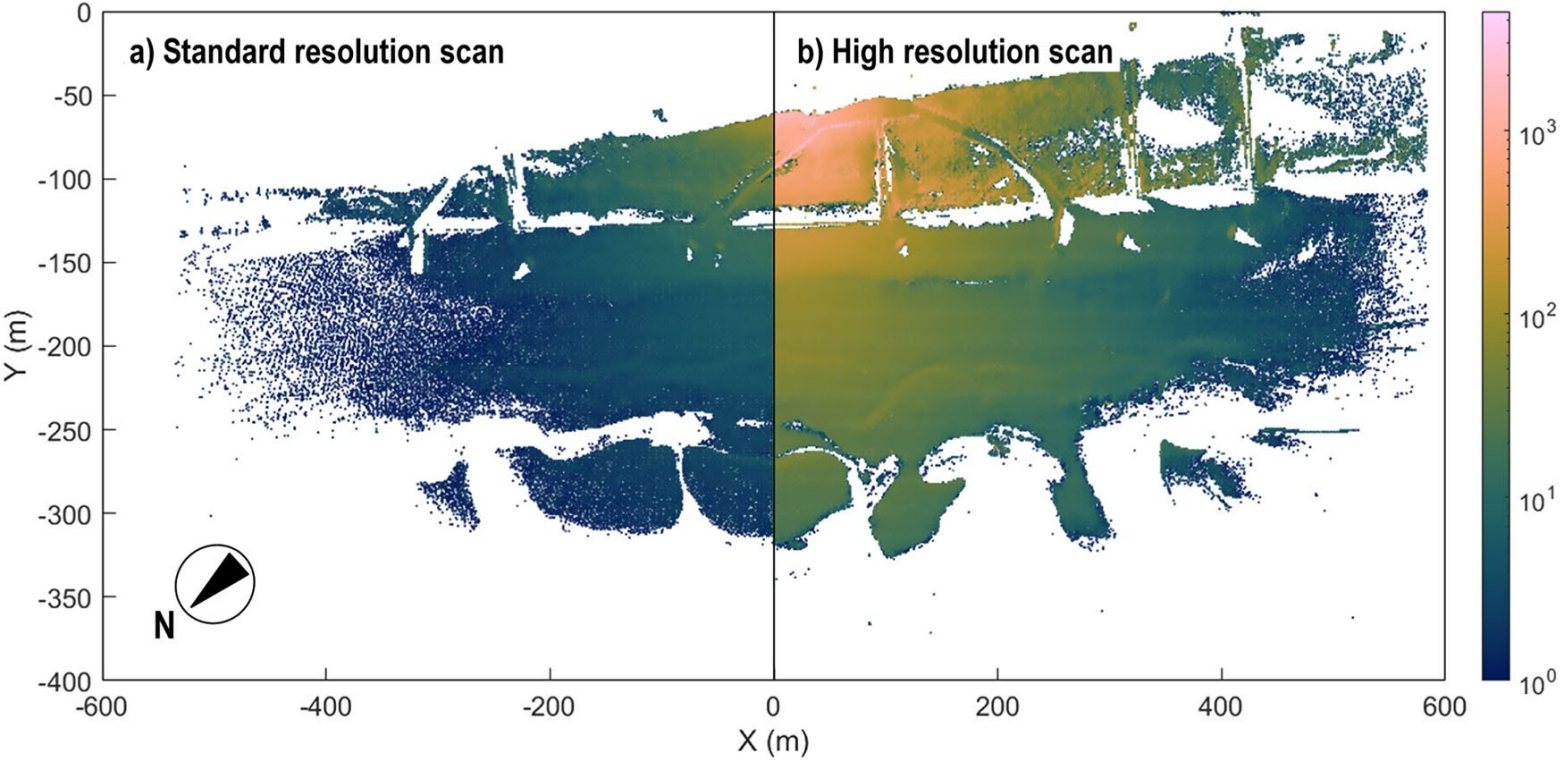
Point densities of a standard- and a high-resolution scan. Displayed point clouds were both acquired on February 1^st^ 2017, around noon, with an angular resolution of 0.05° (**a**) and 0.013° (**b**), respectively. The number of points per square metre decreases with increasing range from the scanner. Gaps in the data are due to occlusion in the scene geometry and/or laser shot dropouts on wet surfaces.

The point density varies spatially within the scene and over time, depending on the measurement geometry, surface reflectance properties, tidal level and meteorological conditions. Typical point densities (see Fig. 6) for a scan vary between 50 (standard resolution) to 4,500 (high resolution) points per square metre on top of the dunes directly in front of the hotel, to 1 to 5 points per square metre in the outer areas of the point cloud scene. Gaps in the point cloud scene are the result of scan occlusions, for example at the foot of the dune and laser shot dropouts in areas of moist to wet surfaces^49^.

Generally, scene coverage is lower when tides are high and larger parts of the outer beach are covered with water. The general horizontal tidal range is between 50 and 75 meters depending on local weather, tide and wave conditions.

## Technical Validation

The acquired point cloud data was validated against four reference datasets: three GNSS surveys (acquired on January 26^th^ ^50^, and February 19^th^ and 24^th,^ 2017), and an airborne laser scanning (ALS) point cloud, acquired on January 21^st^, 2017, by Rijkswaterstaat^51^. For this assessment, the respective epochs from the PLS data were georeferenced using the global transformation matrix (see Methods). PLS and ALS point clouds were manually cleaned for outliers and objects in the scene, so that only ground surface points were compared. Reference data were compared to the temporally nearest low-water scan resulting in a maximum time difference of three hours between the scan time and the GNSS and ALS acquisition, respectively. For the comparison, the vertical difference was derived between either GNSS or ALS point to the horizontally closest point in the respective PLS point cloud within a maximum distance of 50 cm.

**Fig. 7:**
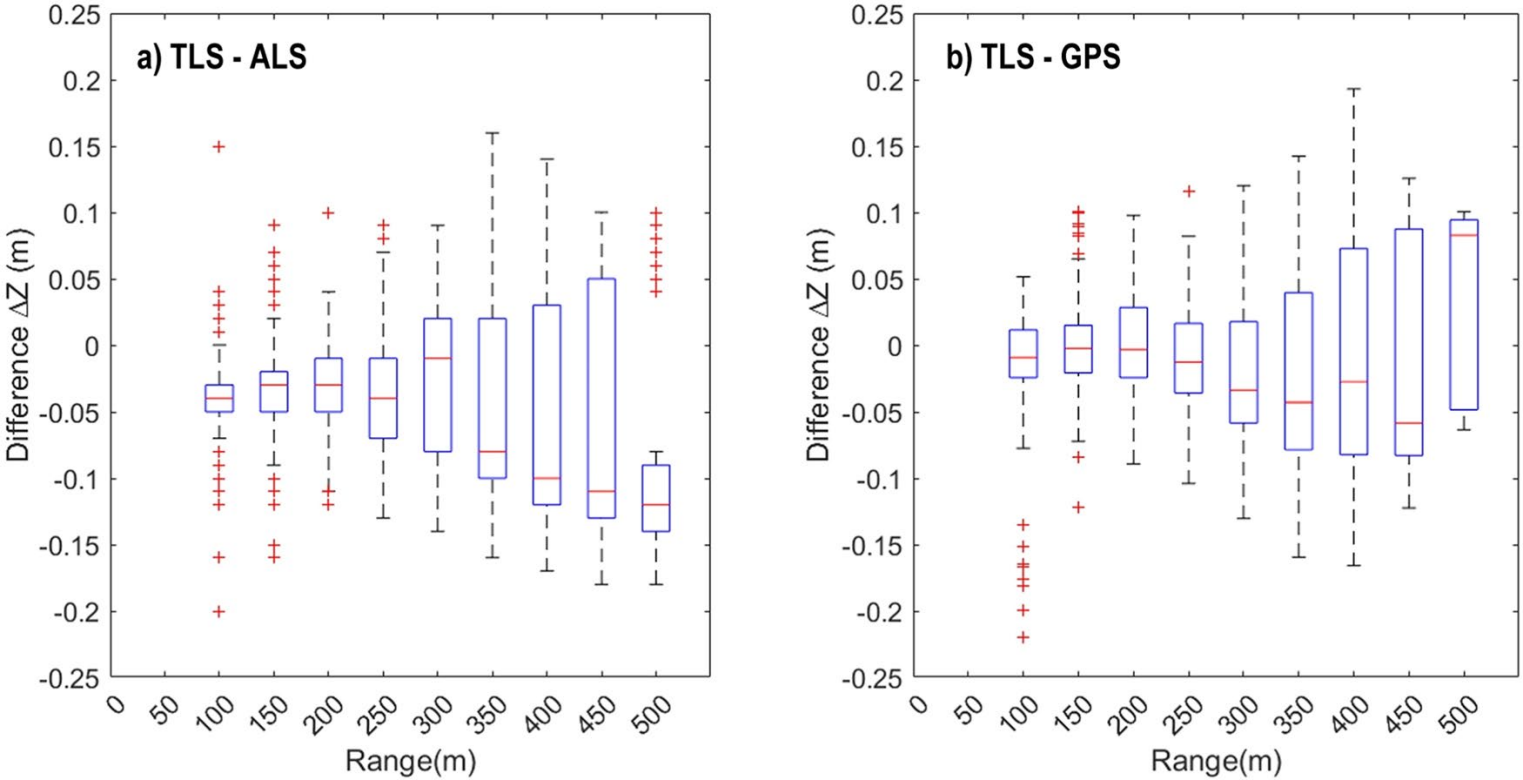
Comparison of terrestrial laser scanning (TLS) heights against available reference datasets acquired by airborne laser scanning (ALS) and GNSS. (**a**) Boxplot of the height difference between the TLS and ALS measurements as a function of the TLS scan range. The box indicates the first quartile, median (red line) and third quartile of values with minimum and maximum indicated by the error bar. Outliers are indicated by red points. (**b**) Boxplot of the height differences between the TLS-based height and GNSS measurements of the surface as a function of scan range.

The differences between the acquired point cloud data and the reference data in dependence of the ranging distance to the PLS sensor are shown in Fig. 7. The graphs give an indication of the increasing uncertainty of PLS measurements as a function of the measurement range. The average difference increases by about 1 to 2 cm per 100 m range increase. This performance is similar to results obtained by PLS acquisitions in Belgium with the same PLS setup^52^ and as reported in earlier research^53^.

The uncertainty of the PLS dataset was further assessed regarding the time-dependent alignment between point clouds^32–34^. Although the laser scanner was fixed in a permanent position during the entire observation period, slight movement of the instrument under variable weather influences might occur and affect the time-dependent alignment of topographic measurements. The internal inclination sensor of the laser scanner recorded roll and pitch values with standard deviations of 0.01° during a scan acquired under calm weather conditions. As expected from the permanent observation set up, larger deviations occur during stormy weather conditions (i.e. wind speeds of 10 m/s and higher), amounting to standard deviations of up to 0.1°, equivalent to an error of about 1.7 cm in height at 10 m range.

Two approaches have been deployed to assess the relative alignment accuracy of the 4D point cloud data: one based on the stability of measured range distances to ground control points and baselines between stable objects^25^ and the other based on point-to-plane distances between stable planar surfaces in the scene^33^.

Range measurements to stable reference objects in the scene are expected to be constant if the laser scanner experienced no movement at all. However, daily and seasonal atmospheric effects of humidity and surface moisture influence the refraction of the laser beam which appear as fluctuations in the range measurements that are indirectly linked to temperature variations^33^. In the assessment of time-dependent ranges^34^, the range measurement to three reference objects in the scene at a distance of 80 to 360 m to the laser scanner showed variations of 2.0 to 3.0 cm during the six-months period (from November 11^th^, 2016 to May 25^th^, 2017).

The time-dependent measurement uncertainty of acquired point clouds is further examined by comparing the lengths of three baselines between stable reference objects with baseline lengths between 90 and 425 m. These baselines were derived for daily scans during low tide and yielded an average daily standard deviation of 1.0 to 3.0 cm.

The alignment accuracy after application of the time-dependent, rigid transformation per epoch is assessed via point-to-plane distances for each epoch to the reference scan, using an independent set of stable planar surfaces^33^. These time-dependent residual distances for 13 stable surfaces in the scene yield a median alignment accuracy of 0.4 cm with a standard deviation of 1.9 cm for the entire time series.

These assessments of time-dependent alignment provide an estimate of the uncertainty to be considered in analysing the 4D point cloud data, particularly the minimum change that can be confidently detected between two or more point clouds in the time series.

## Usage Notes

Typical usage of topographic point clouds regards the derivation of morphometric properties of the scene, for example by deriving a Digital Elevation Model (DEM) per epoch^23^. Analyses of temporal dynamics can be performed by comparing the topography between pairs of point cloud epochs. This can be achieved via DEM differencing^54^ or via direct point cloud comparison^55^. Coastal studies have further made use of backscatter information, i.e. the laser return intensity, to analyse spatially and temporally variable surface moisture on the beach^56–58^.

Point clouds can be processed, visualised and corrected with various open source tools (e.g., CloudCompare^39^, Point Cloud Library^59^ or Python with the laspy package^60^) and closed source software packages for point cloud processing (e.g., Matlab^61^, LAStools^62^ and OPALS^63^).

**Fig. 8:**
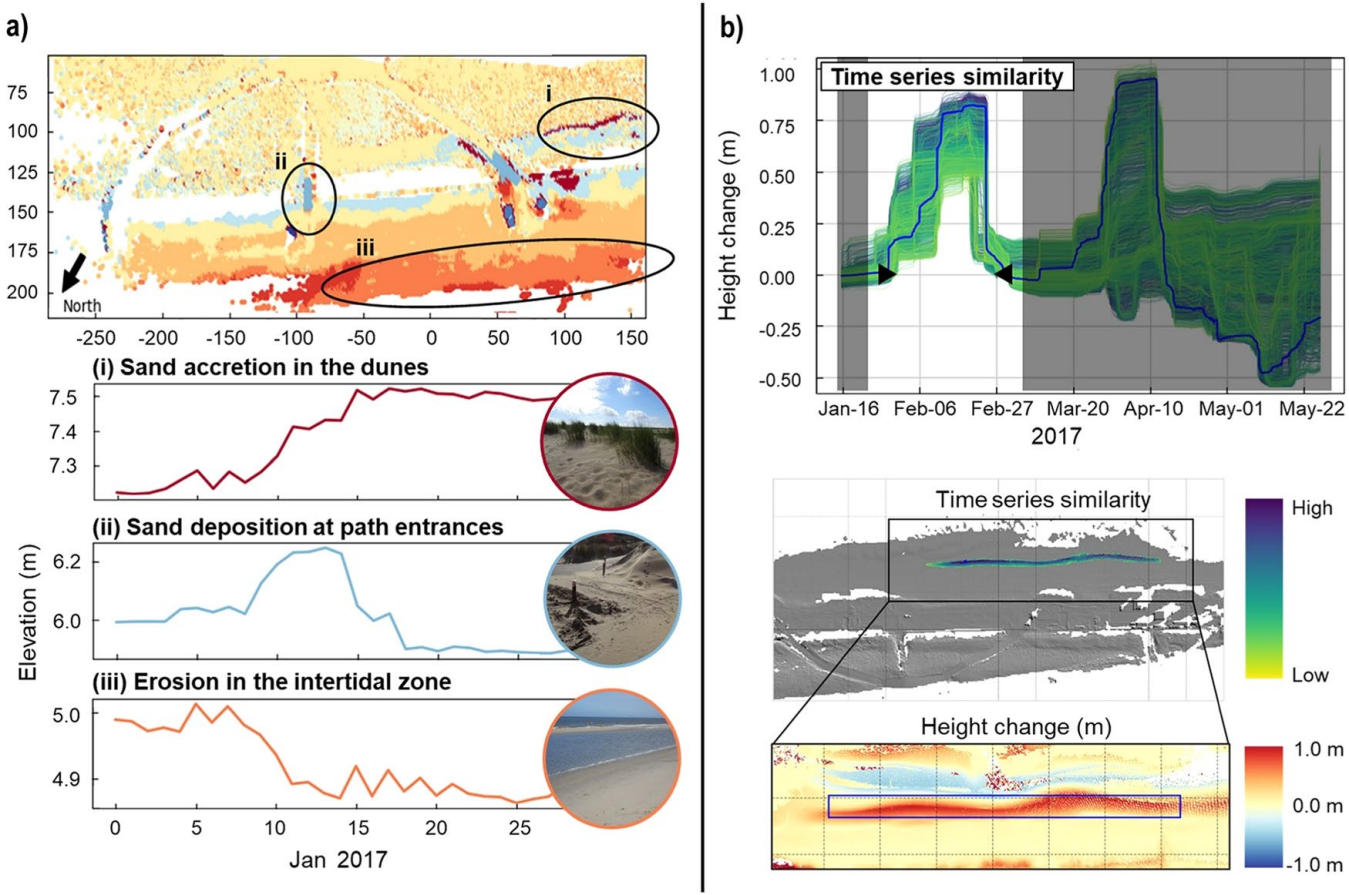
Time series-based methods for change analysis applied to 4D point clouds of Kijkduin. (**a**) Time series clustering to identify change patterns in the beach scene. Subfigures show an example of identified sand deposition in small areas where the paths meet the beach and erosion in the intertidal area for daily point clouds of January 2017. (**b**) Spatiotemporal segmentation to extract change forms such as temporary accumulation through a sandbar. Change forms are spatially delineated regarding similar histories of surface change in neighbouring locations during their occurrence in the time series (period outside change form shown greyed).

To make use of the full temporal domain in 4D point cloud data, first methods were developed to extract information on coastal surface processes. Time series clustering^64^, using time series at locations in a grid of 1.0 m resolution as input, was used to identify change patterns in the beach-dune scene, by grouping areas on the beach/dune that are subject to similar surface change dynamics, such as continual erosion in the intertidal zone or sand deposition at anthropogenic infrastructure (Fig. 8(a)). To automatically detect and extract individual temporary changes from full 3D time series, a method of spatiotemporal segmentation was developed^65^. This time series-based method removes the need to select analysis periods for change analysis, as temporary accumulation and erosion are detected and delineated using the surface change histories of locations in the scene (Fig. 8(b)).

Methodological research to enable automatic analysis of 4D point cloud data is ongoing, and may benefit from the dataset presented herein. Resulting information on surface change dynamics can be used for subsequent analysis of coastal processes at this site.

External meteorological and hydrodynamic data are available to integrate in the analysis of coastal behaviour at Kijkduin. The Dutch meteorological office (KNMI) provides weather information of Hoek van Holland^66^, the closest professional weather station to Kijkduin. Rijkswaterstaat^67^ provides wave height information on the North Sea. These additional meteorological and wave height data are provided with the scan dataset on Pangaea^44^, with friendly permission from KNMI and Rijkswaterstaat. Additionally, local wave information modelled with Delft3D-Wave can be integrated, for which the data and scripts are openly available as supplement to^25^.

## Data Availability

The transformation matrices for time-dependent alignment were calculated using the module ICP in the software package OPALS^62^. The rigid alignment was calculated by minimizing point-to-plane distances using a search radius of 0.5 m for plane fitting and a sampling distance of 0.05 m. Stable parts were extracted with 0.5 m radius at locations of planar surfaces. Python and Matlab scripts are provided with the data, with basic functions to read and write the point cloud data from the LAZ files, to apply the rigid transformation matrices to the point cloud data for time-dependent alignment and georeferencing, and to read out information on scan settings from the metadata files of an epoch.
